# Prevention and Treatment of HPV-Induced Skin Tumors

**DOI:** 10.3390/cancers15061709

**Published:** 2023-03-10

**Authors:** Daniel Hasche, Baki Akgül

**Affiliations:** 1 Division of Viral Transformation Mechanisms, Research Program “Infection, Inflammation and Cancer”, German Cancer Research Center (DKFZ), Im Neuenheimer Feld 242, 69120 Heidelberg, Germany; 2 Institute of Virology, Medical Faculty and University Hospital Cologne, University of Cologne, Fürst-Pückler-Str. 56, 50935 Cologne, Germany

**Keywords:** papillomaviruses, HPV, NMSC, cSCC, vaccination

## Abstract

**Simple Summary:**

Non-melanoma skin cancer is the most common cancer in humans and has been linked to skin infections with betaHPV. This has led to the development of vaccine candidates against these viruses. This review provides an overview of the currently followed prophylactic and therapeutic vaccination strategies.

**Abstract:**

Non-melanoma skin cancer (NMSC) is the most common cancer in humans with increasing incidence. Meanwhile, a growing body of evidence has provided a link between skin infections with HPV of the genus beta (betaHPV) and the development of cutaneous squamous cell carcinomas (cSCCs). Based on this association, the development of vaccines against betaHPV has become an important research topic. This review summarizes the current advances in prophylactic and therapeutic betaHPV vaccines, including progresses made in preclinical testing and clinical trials.

## 1. Introduction

Cutaneous squamous cell carcinoma (cSCC) of the skin is the most common metastatic skin cancer and its incidence is increasing worldwide. cSCC is a progressed form of premalignant actinic keratosis (AK) that occurs on sun-exposed body areas. Continuous UV damage caused by chronic sun exposure is known to be the main cause of cSCC development [1]. In recent years, it has become increasingly evident that human papillomaviruses (HPV), apart from chronic UV irradiation, immunosuppression and genetical predispositions, represent an important co-factor for cSCC development. HPVs are small non-enveloped epitheliotropic DNA viruses with a circular genome of approximately 8000 bp. At present, 441 HPV types are known to have co-evolved and to persist in the human population. HPVs are phylogenetically grouped into five genera according to DNA sequence homology in the L1 gene (encoding the L1 capsid protein), namely alpha (66 types), beta (67 types), gamma (302 types), mu (5 types) and nu (1 type) (http://pave.niaid.nih.gov/ (accessed on 12 January 2023); [2,3]). AlphaHPV types infect both cutaneous and mucosal epithelia, and are further classified as either low-risk or high-risk HPV (HR-HPV) [4]. The critical roles of HR-HPVs, most notably HPV16 and HPV18, is now well established in the etiology of cervical cancer and is supported by a wealth of epidemiological and experimental data [5]. These types are also implicated in the majority of other anogenital cancers [6] as well as oropharyngeal squamous cell carcinoma [7,8,9]. Cutaneous HPVs are distributed across all five HPV genera with the gammaHPV genus being the most diverse and largest clade within the family *Papillomaviridae* (pave.niaid.nih.gov, accessed on 12 January 2023; [2]). The association between betaHPV types and non-melanoma skin cancer (NMSC) development was initially described in patients with *Epidermodysplasia verruciformis* (*EV*), an autosomal recessive predisposition where patients develop cSCCs mainly on sun-exposed body sites [10,11,12]. The consistent finding of betaHPV types in EV-associated cSCC suggests betaHPVs as etiologic agents for cSCCs; it is also worthwhile and important for this link to be studied in non-EV patients [13,14]. In EV-associated cSCCs, a multiplicity of betaHPV types, especially HPV5 and HPV8, are found [15,16] that can also be linked with the development of actinic keratoses and cSCC in patients of the general population [17,18,19,20]. Molecular and functional studies of betaHPV oncoproteins E6 and E7 have demonstrated their negative effects on skin homeostasis. They play a critical role in the viral life cycle by disrupting epithelial differentiation and immune homeostasis, promoting cell proliferation and expanding the epithelial progenitor cell compartment to ensure viral DNA replication and progeny production. However, they also inhibit UV-induced DNA-damage repair and apoptosis and thereby enhance the mutagenic capacity of UV exposure [12,18,21,22,23]. Consequently, infected cells may accumulate a higher number of genomic mutations, contributing to the development and progression of AKs and cSCC.

In this article, we present an overview of the advancements in vaccine development that may prevent the occurrence of betaHPV-associated skin tumors and describe novel immunotherapeutic approaches for the treatment of virus-induced tumors.

## 2. Economic Burden of cSCC

cSCC accounts for 20–50% of all skin cancers [24]. The incidence rates of NMSC and cSCC are constantly increasing in the Caucasian population. In Germany, the incidence of NMSC among people with statutory health insurance increased by 53% between 2009 and 2015, a ~7% increase per year [25], with more than 500,000 new NMSC cases reported in 2019 (TK Skin Cancer Report 2019, [26]).

In the Netherlands, where 145,618 patients received a diagnosis of a first cSCC between 1989 and 2017, cSCC incidences increased substantially between 2002 and 2017 (8.2% per year female patients and 5.7% for male patients) [27]. In Ontario, Canada, an average annual percentage rise in cSCC of ~1.9% was calculated for the time period of 2008 to 2017 [28]. In Japan, the incidence rate of cSCC even quadrupled between 2007 and 2016 [29].

Although mortality may be relatively low, the direct and indirect costs of NMSC (including excisions, follow-up care, radical lymph node dissection, cryotherapy and radiation therapy [30]) represent a significant challenge for health systems. As analyzed by a market research group, the global NMSC market (including 16 countries) reached USD 504.6 million in 2021 and is expected to increase by 5.5% to USD 692.9 million until 2027 [31]. For example, in Canada, the combined direct and indirect costs of 1710 cSCC cases attributable to occupational solar radiation exposure (representing 9.2% of all 18,549 newly diagnosed cSCC cases in 2011) were USD 13 million plus additional intangible costs of USD 5.1 million, which amounts to a total cost of USD 10,555 per case [32].

In the US, considering nearly 16,000 cSCC patients between 2009 and 2015, the costs per cSCC even reached USD 60,841 due to an average hospitalization of ~6 days [33].

Considering Europe, between 2015 and 2018, Italy annually spent an estimated EUR 25.9 million for management and treatment of patients with cSCC, out of which EUR 2.7 million were associated with advanced cSCC [34]. The average cost per cSCC patient was EUR 2236, whereas it was more than twice as high for patients with advanced cSCC [34]. The estimated total cost in just one single Spanish Hospital for treating 3163 NMSC patients between 2006 and 2010 reached nearly EUR 3.4 million, with individual expenses ranging from EUR 423 (minor outpatient surgery) to EUR 1832 (inpatient surgery) [35].

According to an estimation, South Africa annually spends USD 13.8 million for treating NMSC at individual costs of USD 470 per cancer case [30]. In Australia, NMSC treatments increased by 86% between 1997 and 2010, and costs for NMSC care (including diagnosis, treatment and pathology) were estimated to exceed AUD 700 million in 2015 [36]. UV light, as an environmental risk factor, is the main cause of skin cancer, and more than 80% of these lesions appear at sun-exposed body sites [37]. The generally discussed climate change leads to more extreme sun exposure worldwide, which consequently has an impact on skin cancer risk and may further increase both incidence and resulting costs for the health systems.

## 3. The Impact of Immune Suppression

After initial infections in early childhood, the risk of cSCC increases sharply with age, possibly in addition to the accumulation of UV damage in the skin as a result of immune senescence in the elderly. In immunocompetent individuals, persistent betaHPV infections are tightly controlled by the immune system and usually asymptomatic. In contrast, there are even indications that the presence of cutaneous HPVs in the skin may be protective. A recent study by Strickley et al. showed that infection with mouse papillomavirus 1 (MmuVP1) [38] prior to chemically or UV-induced carcinogenesis suppressed skin cancer development in mice [39]. Although controversially discussed [40], this study led to the hypothesis that the presence of cutaneous HPV types may even have an evolutionary benefit to humans by protecting them from the mutagenic components of UV light. However, infections can be problematic in iatrogenically immunosuppressed patient groups, particularly organ transplant recipients (OTRs) [17]. The amount of betaHPV DNA in the skin of healthy individuals varies over seven orders of magnitude and is considerably increased in immunosuppressed individuals. Weakened immune control directly and significantly increases skin cancer risk by 100- to 200-fold, which often results in rapidly progressing field cancerization at sun-exposed body sites, causing high morbidity and mortality [41]. This is consistent with the assumption that immunosuppression leads to higher betaHPV loads and increased oncogenic activity, which in turn increases the risk of cSCC development [17,21,42].

Despite the significantly increased incidence of cSCC, immunosuppressed patients do not differ significantly from immunocompetent individuals with respect to the betaHPV-type spectrum [43,44]. However, a significantly higher proportion of immunosuppressed patients harbor the highest betaHPV DNA loads in plucked eyebrow hairs. This significantly higher proportion of immunosuppressed patients may indicate the importance of poorly controlled betaHPV infection in skin carcinogenesis [17,42,45]. An estimated 40% of OTRs will develop NMSC within the first 10 years following transplantation and up to 80% after up to 20 years [41], with only 56% of 3-year disease-specific survival [46]. Furthermore, the overall survival rate of patients with metastatic cSCC over 10 years is less than 20% [47]. Therefore, this high-risk patient group may benefit from anti-betaHPV vaccines.

Interestingly, the mechanism of betaHPV-dependent carcinogenesis appears to be different in immunocompetent individuals than that in immunosuppressed patients, since the viral loads tend to be higher in AK than in cSCC. In contrast to alphaHPV-associated cancers, in which HPV DNA is detectable in tumor cells, even at advanced stages, the episomally persisting viral DNA is lost in progressive stages of cSCC in immunocompetent patients [18,48]. Higher betaHPV DNA loads in AK are consistent with the oncogenic activity of cutaneous HPV in the early phases of skin cancer development. It can be speculated that, starting from very low basal oncogene expression, a threshold level of oncogene expression must be exceeded in very well-controlled persistent betaHPV infection to induce skin tumors [49]. Taken together, given that current HPV vaccines do not provide coverage for betaHPVs, novel vaccines are needed to prevent infection-related cSCC in high-risk patient groups.

## 4. Area of Concern: What Are the “High-Risk” Cutaneous HPV Types?

Epidemiological studies have greatly aided in defining the causal role of high-risk alphaHPV types in anogenital and oropharyngeal cancers. Most epidemiological studies have also focused on stratifying the risk of developing AK and cSCC linked with specific betaHPV types. However, in contrast to alphaHPVs, a broad spectrum of betaHPVs is frequently detected in cSCC, with no specific types dominating. In other words, the “HPV16 or HPV18 of skin equivalent” is unknown and may not even exist. In contrast to alphaHPV types, humans are most likely to be infected with betaHPVs in the weeks following birth. A variably extensive commensal betaHPV flora rapidly develops on the skin and is composed of frequently exchanged (transient) and long-persisting betaHPV types [50,51]. There is also some evidence of individual susceptibility to certain HPV types, which may define the persistent types that are important in promoting cancer development [52,53,54,55].

This would mean that the persisting betaHPV type on the skin of individuals may represent “the” oncogenic HPV type for this person. Therefore, if a pan-betaHPV vaccination strategy is not feasible, skin swabs would need to be collected from the patient over an extended period of time to identify the persistent betaHPV types to specifically target them. This would lead to an individual vaccine cocktail for each patient, making the entire approach laborious and costly.

Another issue is that the relationship between AK and cSCC with gammaHPV infections has not been examined in detail. Bolatti et al. recently reported a higher HPV prevalence and viral load in AK than in cSCC and detected an even higher prevalence of gammaHPV in AK than betaHPV and alphaHPV types [56]. Therefore, one should not exclude the role of gammaHPV in skin tumorigenesis. Consequently, it is likely that it will not be possible to define the high-risk cutaneous HPV types and that many cutaneous types may play a role in tumorigenesis. As multiple betaHPV types are potentially involved in the development of cSCC, the treatment of high-risk patients would ideally require a broadly protective vaccine to prevent viral amplification.

## 5. Area of Concern: When Should a Prophylactic Vaccine Be Administered?

**Figure 1:**
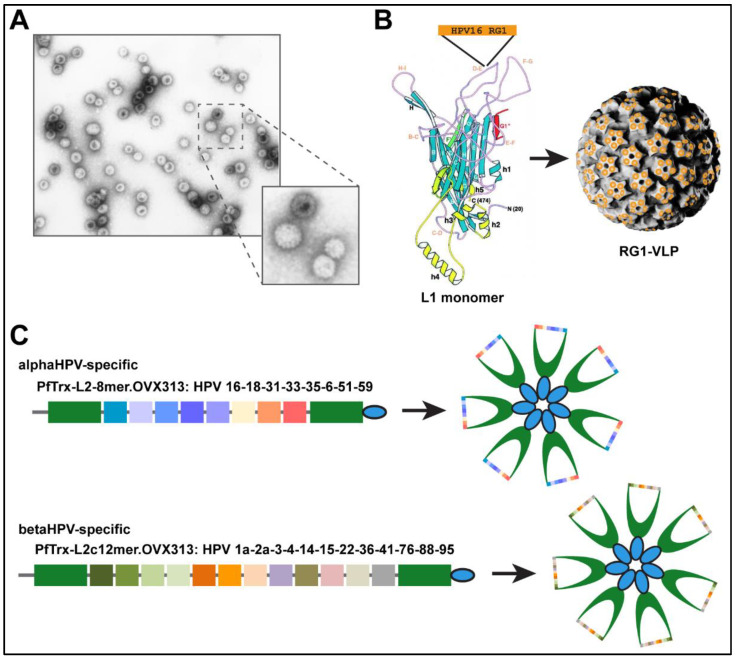
HPV vaccination strategies. (**A**) Electron micrograph of L1 VLPs. (**B**) In HPV16VLP-HPV16RG1, the HPV16 L2-RG1 (aa 17–36) is inserted into the D-E surface loop of the HPV16 L1 monomer, creating a chimeric fusion protein. Helices (h1–h5) and loops (B-C, H-I, D-E, F-G, C-D, E-F) of the L1 protein are labeled. After self-assembly into VLPs, the RG1 epitope is exposed 360 times [modified from [63]]. (**C**) In PfTrx-based vaccines, the L2 peptides aa20-38 from the various HPV types are inserted into the thioredoxin scaffold (PfTrx, shown in green) derived from the thermophile archaea *Pyrococcus furiosus*. The OVX313 domains (shown in blue) assemble to heat-stable heptamers, leading to a seven-fold presentation of the respective PfTrx-L2 in the PfTrx-L2-8merOVX313 or the PfTrx-L2-c12merOVX313 fusion protein (adapted from [63]).

Infection with alphaHPVs typically occurs shortly after onset of sexual activity. To prevent these infections and the development of HPV-associated diseases, many countries now recommend vaccination before sexual debut. The currently licensed HPV vaccines Cervarix^®^, Gardasil^®^ and Gardasil^®^ 9 are composed of the L1 major capsid protein of HPV, which has the ability to self-assemble into highly immunogenic repetitive virus-like particles (VLPs), exposing the same L1 epitopes 360 times on their surface [57] (Figure 1A). However, they are restricted to type-specific prevention and are directed against mucosal HPV types associated with anogenital and oropharyngeal cancer. They confer near 100% protection against the emergence of HPV16- or HPV18-induced cervical neoplasia (precursors of cervical cancer), and in the case of Gardasil^®^, also against HPV6- and HPV11-induced lesions. Gardasil^®^ 9 additionally covers HPV31, 33, 45, 52 and 58, which, together with protection against HPV6, 11, 16 and 18, confers protection against anogenital, head, neck and precancerous lesions. The L1-based VLP vaccines against alphaHPV types have a prophylactic effect and are not licensed for the treatment of existing infections and tumors. The main mechanism of action of these vaccines is the induction of high-titer neutralizing antibodies that provide type-restricted protection against vaccine-induced HPV infections.

BetaHPV antigens are not included in the licensed VLP-based HPV vaccines; therefore, no standardized vaccine against betaHPVs is licensed to induce neutralizing antibodies against viral capsid proteins. Given the remarkable success of vaccines against alphaHPV types, the field is now focusing on developing similar VLP-based prophylactic strategies for AK and cSCC caused by cutaneous HPV [58]. A universal VLP-based vaccine covering all known betaHPV types, with a multivalent VLP-based vaccine, represents a technical challenge. However, a feasibility study was performed using the natural preclinical *Mastomys coucha* model [18] to test whether VLP-based vaccines may also protect against cutaneous papillomavirus-induced skin tumors. This African multimammate mouse is the natural host of the cutaneous *Mastomys natalensis* papillomavirus (MnPV) [59] and allows for the monitoring of serological responses against the virus during skin tumorigenesis [60,61,62]. Vaccination with a MnPV-specific VLP-based vaccine fully protected *Mastomys coucha* against experimental infection and associated skin tumors in both healthy and immunocompromised animals [60]. The protective effect results from the antibody-dependent prevention of viral spreading, keeping the viral load in the skin low. Thus, tumor formation could be prevented, even in animals that were already infected at the time of vaccination. These results were a proof-of-concept for the feasibility of vaccination against cutaneous papillomaviruses and demonstrated the high value of a VLP-based prophylactic vaccine against cutaneous HPV. Considering that these results originate from animal studies with one particular virus type, the clinical translation described in the next chapter will demonstrate its practicability in humans.

## 6. Prophylactic Vaccine Strategies in Clinical Trials

Several clinical trials are currently underway to test the practicality of the different approaches. Considering their limited ability to confer cross-protection, it is likely impractical to use genotype-specific L1-VLP vaccination for cSCC, as there are more than 60 betaHPV types with no clear subset linked to cSCC. Thus, the focus has shifted to the development of next generation L2-based vaccines with broader protective efficacy [18]. The HPV L2 protein is suitable for this purpose because it contains a region that is well conserved between mucosal and cutaneous HPVs and serves as an important cross-neutralizing epitope [64,65]. A drawback of L2-based vaccines, however, is their lower immunogenicity and significantly lower capability to induce neutralizing antibody titers compared to L1-based vaccines [66]. Consequently, a simple HPV16-L2 DNA vaccine failed to induce such neutralizing antibodies [59]. The main goal is to develop a single or oligovalent antigen that provides higher antibody titers and a much broader spectrum of protection against HR and cutaneous HPV types [64]. Several L2-based vaccination strategies already showed promising results of being able to successfully induce a broad spectrum of cross-neutralizing antibodies. One such approach involves the use of chimeric VLPs. Here, the conserved RG1 epitope of L2 (residues 17–36, named after the monoclonal antibody RG1 binding in that region) is incorporated into the immunodominant D–E surface loop of HPV16 L1-VLPs and displayed on the surface of assembled VLPs [67,68] (Figure 1B). The so called HPV16VLP-HPV16RG1 looks like a promising vaccine in a preclinical study, inducing both cross-neutralizing antibodies to twenty other mucosal HPVs and five betaHPV types (HPV5, 20, 24, 38 and 96), as well as strong cytotoxic T cell responses together with high antibody titers to HPV16. Since the additional RG1 epitope does not negatively affect L1-specific immune responses [69], this vaccine combines high HPV16-specific with broad-spectrum L2-based immunogenicity. This vaccine candidate is currently being tested in a multicenter Phase I study [65]. Moreover, RG1 VLPs against cutaneous HPVs, containing the consensus RG1 sequences derived from multiple cutaneous HPV types in the D–E loop of VLPs, have recently been preclinically tested. This increases the potential of broadly cross-neutralizing antibodies as well as cytotoxic T cell responses against skin-specific HPV types [70].

Another vaccine approach is based on a fusion protein of human calreticulin (hCRT) and HPV16-E6/E7/L2 and, after cGMP (current good manufacturing practice) production by the NCI PREVENT program [71], has recently entered clinical Phase I [65]. The fusion protein enhances MHC class I presentation and induction of both E6/E7-specific T-cell responses and L2-specific neutralization, thereby indicating prophylactic and therapeutic potential [72,73]. However, so far this approach has not yet been tested in the context of cutaneous HPV types.

A further promising approach, which is already produced under cGMP conditions, is currently entering clinical Phase I testing. Here, a fusion of multimeric polytopes, comprising eight different mucosal HPV L2 (residues 20–38) into the thermo-resistant bacterial thioredoxin scaffold protein (Trx) of the archaea *Pyrococcus furiosus* (Pf), leads to the generation of the synthetic PfTrx-8mer antigen, which significantly increases cross-reactivity [74,75]. Interestingly, the fusion of the OVX313 oligomerization domain with the *Pf*Trx-8-mer antigen further improved this antigen [76], inducing neutralizing antibodies against the incorporated mucosal types and all cutaneous HPVs tested (types 3, 4, 5, 10, 38, 63, 76, 92, 95 and 96) in a preclinical study using guinea pigs [77] (Figure 1C). In contrast to VLP-based vaccines, the construction of the antigen makes the vaccine temperature insensitive while inducing a broad and robust cross-neutralizing antibody response [77]. Recently, Mariz et al. successfully tested the cutaneous HPV-specific vaccine candidate *Pf*Trx-L2c12mer-OVX313 covering HPV types 1a, 2a, 3, 4, 14, 15, 22, 36, 41, 76, 88 and 95, respectively, in a preclinical study and demonstrated broadly neutralizing immune responses against 19 cutaneous HPVs in guinea pigs [78] (Figure 1C).

Data on the efficacy of L2-based vaccines for preventing skin tumors—the ultimate read-out of a successful immunization—in preclinical models are generally lacking. An important question is therefore whether vaccination can induce sufficient (cross-)neutralizing antibody titers to confer protection against tumor formation. In this regard, the *Mastomys coucha* model was recently used in an exploratory study to test the cross-protection of vaccination with HPV16VLP-HPV16RG1 and betaHPV-specific PfTrx-L2-c.12merOVX313 against experimental MnPV infection. Notably, both vaccine candidates were able to induce cross-neutralizing antibodies and offered cross-protection against MnPV-induced skin tumors [63].

Taken together, these vaccine candidates, if administered to patients before the onset of immunosuppression, may in fact provide protection against virus-induced skin tumors in men.

## 7. T-Cell Mediated Approaches against HPV-Induced Lesions

Considering the multiplicity of betaHPV and gammaHPV types without clearly defined etiology in their clinical manifestations, the question arises whether prophylactic vaccination should be recommended. We still do not know what allows some AKs to progress to cSCC and why only some respond to treatment. Studies comparing various treatments have been published; however, no gold-standard treatment has been established to date [79]. Topical agents are the most commonly used field-targeted therapies, and several agents are available and approved for treatment of AK, including 5% imiquimod cream (Aldara^®^), a TLR7 agonist. Since the recurrence rate after treatment is high and the success rate of Aldara^®^ treatment is only about 50% [80], there is still a strong need for better therapies [81]. Focusing on the betaHPV load on successfully treated patients—before and after treatment—may help to understand whether the key for the success of the therapy by currently approved treatments is mediated by an HPV-specific immune response. Notably, a few case studies described the regression of cutaneous lesions following vaccination with the licensed VLP-based vaccines [82]. Nevertheless, there are also conflicting reports stating low or even no benefit, and randomized controlled studies are completely missing.

However, since there is increasing evidence of a direct link between HPV and AK, treatment strategies targeting viral oncoproteins could be important in the prevention of AK and their potential progression to cSCC. Thus, a vaccination strategy capable of activating a strong T cell-mediated immune response, with the capacity to recognize and to eliminate skin cells expressing high levels of HPV early proteins, may be considered as a therapeutic option to treat skin tumors and prevent recurrence.

The therapeutic focus here is on E6 and E7 as antigenic targets [83]. To test different vaccination approaches against betaHPV early proteins, K14-HPV8-CER transgenic mice were used. In this preclinical model, the complete early genome region of HPV8, without the capsid proteins L1 and L2, is expressed in the skin under the control of the human keratin-14 (K14) promoter [84]. Viral antigens are synthesized at low levels, and this sub-threshold expression does not induce tumor formation per se. This is comparable to the situation in immunocompetent humans where betaHPV loads are also low, only leading to well-controlled asymptomatic infections. In this experimental system, UV irradiation or mechanical skin wounding activates higher HPV8 early gene expression, resulting in skin tumor formation within three weeks [49,85,86]. This parallels the situation in immunocompromised patients with higher viral loads and skin cancer incidence. Skin tumor development is always preceded by enhanced viral oncogene expression already one to two days after UV irradiation or wounding [49]. Hence, an early increase in oncogene expression turned out to be crucial for tumorigenesis, as the transient siRNA-mediated knockdown of E6 mRNA led to lower papilloma incidence [49].

To generate an animal model for immunotherapy of HPV8-induced skin tumors, HPV8-CER transgenic skin was transplanted onto non-transgenic littermates. The grafted HPV8 transgenic skin was not rejected by the recipient’s immune system and always formed papillomas. For immunoprevention trials, wild-type FVB/n mice were immunized by delivering expression vectors for HPV8-E6 into the epidermis before transplantation. Successfully vaccinated animals did not reject the transplanted HPV8 transgenic skin but also did not exhibit papilloma formation and elicited a cytotoxic T-cell response against HPV8-E6 [87]. This underscores the suitability of the transgenic K14-HPV8-CER mouse as a preclinical animal model to develop immunotherapeutic treatment options for skin with high betaHPV loads.

**Figure 2:**
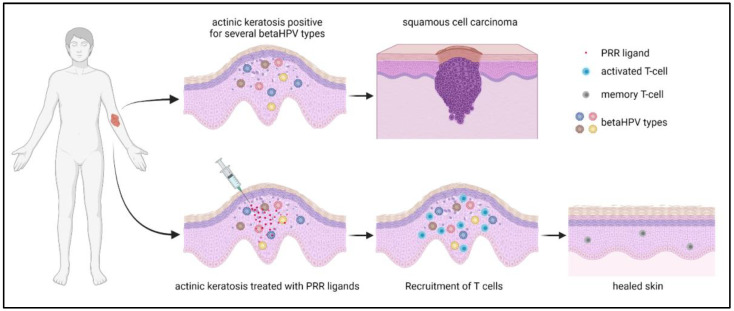
Graphical illustration of an hypothetical activation of an innate immunity-driven in situ autovaccination strategy against the patient’s “own” betaHPV types to induce protective T cell-mediated immunity and to prevent cSCC development (data from [88]).

In a recent proof-of-concept study, the hypothesis of whether the activation of an innate immunity-driven in situ autovaccination strategy against the patient’s “own” betaHPV types can effectively induce protective T cell-mediated immunity was tested (Figure 2). This study focused on the immune therapeutic activation of pattern recognition receptors (PRRs), which, together with high levels of viral early gene expression in K14-HPV8-CER mice, can restore immune activity and prevent skin tumors. Here, different nucleic acid receptor ligands were delivered into the murine skin and tumor formation was subsequently assessed. The tested cGAS/STING, TLR7, 8 and 9 ligands prevented skin tumor development in 25–50% of treated animals. Intriguingly, poly(I:C), a ligand for MDA5 and TLR3, prevented tumor formation in 100% of the mice. These results identified poly(I:C) as the most effective nucleic acid receptor ligand for inhibiting tumor formation. This anti-tumor effect depended on the MDA5-mediated induction of interferon-induced genes. Moreover, T-cell depletion demonstrated a predominant role for CD4+ T cells in tumor prevention [88]. While HPV8 early proteins suppress MDA5-dependent cytokine secretion in vitro [89], poly(I:C) may overcome this inhibitory effect on MDA5 in vivo.

## 8. Conclusions

BetaHPVs, along with UV light, are important co-factors during the early phases of cSCC development, particularly in immunocompromised individuals. Broad-spectrum prophylactic vaccines against these HPV types may reduce the incidence of cSCC in high-risk patients. Various approaches are under development, but further research is necessary to determine which of these vaccination strategies is the best for the individual patient and the clinical context. Therapeutic strategies may target keratinocytes expressing high levels of viral oncoproteins to boost T cell activation. Activation of PRRs such as MDA5 should be also considered as an innovative and promising strategy for tumor-inhibition of betaHPV-related tumors.
